# The Jekyll and Hyde Nature of Tetrazoles in Polymer Science: From Intrinsic Electronic Duality to Programmable Macromolecular Function

**DOI:** 10.1002/anie.8793524

**Published:** 2026-07-30

**Authors:** Meryem S. Akdemir, Hatice Mutlu

**Affiliations:** ^1^ Fachbereich Chemie, Technische Polymerchemie Rheinland‐Pfalzer Technische Universität Kaiserslautern‐Landau (RPTU) Kaiserslautern Deutschland; ^2^ Leibniz‐Institut Für Verbundwerkstoffe (IVW) GmbH Kaiserslautern Deutschland; ^3^ Laboratory of Organic and Macromolecular Chemistry (IOMC) Friedrich Schiller University Jena Jena Germany; ^4^ Helmholtz Institute for Polymers in Energy Applications Jena (HIPOLE Jena) Jena Germany; ^5^ Helmholtz‐Zentrum Berlin for Materials and Energy GmbH Berlin Germany

**Keywords:** functional polymer materials, macromolecular design, multicomponent polymerization (MCP), substitution effects, tetrazoles

## Abstract

Nature encodes stability and responsiveness within single‐molecule frameworks, creating systems that remain structurally persistent while retaining access to activated functional states. Tetrazoles embody this principle in synthetic chemistry by combining aromatic persistence with latent reactivity within a single nitrogen‐rich heterocycle. Their intrinsic “Jekyll–Hyde” duality arises from a polarized electronic structure governed by the substitution pattern, which acts as an electronic switch between adaptive, triggerable, and structurally persistent regimes. Although tetrazoles are widely used in small‐molecule chemistry, they remain comparatively underexplored as electronically programmable motifs in polymer science. Their polarity, ion‐binding ability, and photoreactivity are often exploited individually rather than integrated into a broader structure–function framework. This Minireview presents a unifying concept linking tetrazole substitution patterns to electronic identity and, consequently, to macromolecular function in polymer systems. Monosubstituted tetrazoles enable adaptive acid–base responsiveness, hydrogen bonding, and ionic network formation. In contrast, 2,5‐disubstituted tetrazoles serve as photoaddressable precursors for nitrile imine‐mediated ligation, covalent fixation, and fluorescent readout, whereas regio‐defined 1,5‐disubstituted tetrazoles provide electronically locked, highly polar heteroaromatic motifs for persistent polymer architectures. Thus, the mini‐review examines how synthetic strategy governs the formation and preservation of these substitution‐defined tetrazole motifs during polymer synthesis, establishing practical design principles for rational development of functional polymer materials.

## Graphical abstract

Tetrazoles are reframed as substitution‐programmable heterocycles rather than static functional groups in polymer chemistry. By linking electronic identity to synthetic strategy and polymer architecture, this mini‐review establishes a design framework for translating tetrazole duality into predictable macromolecular function.

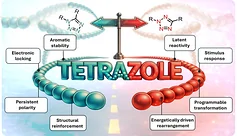

## 1 Introduction

**FIGURE 1:**
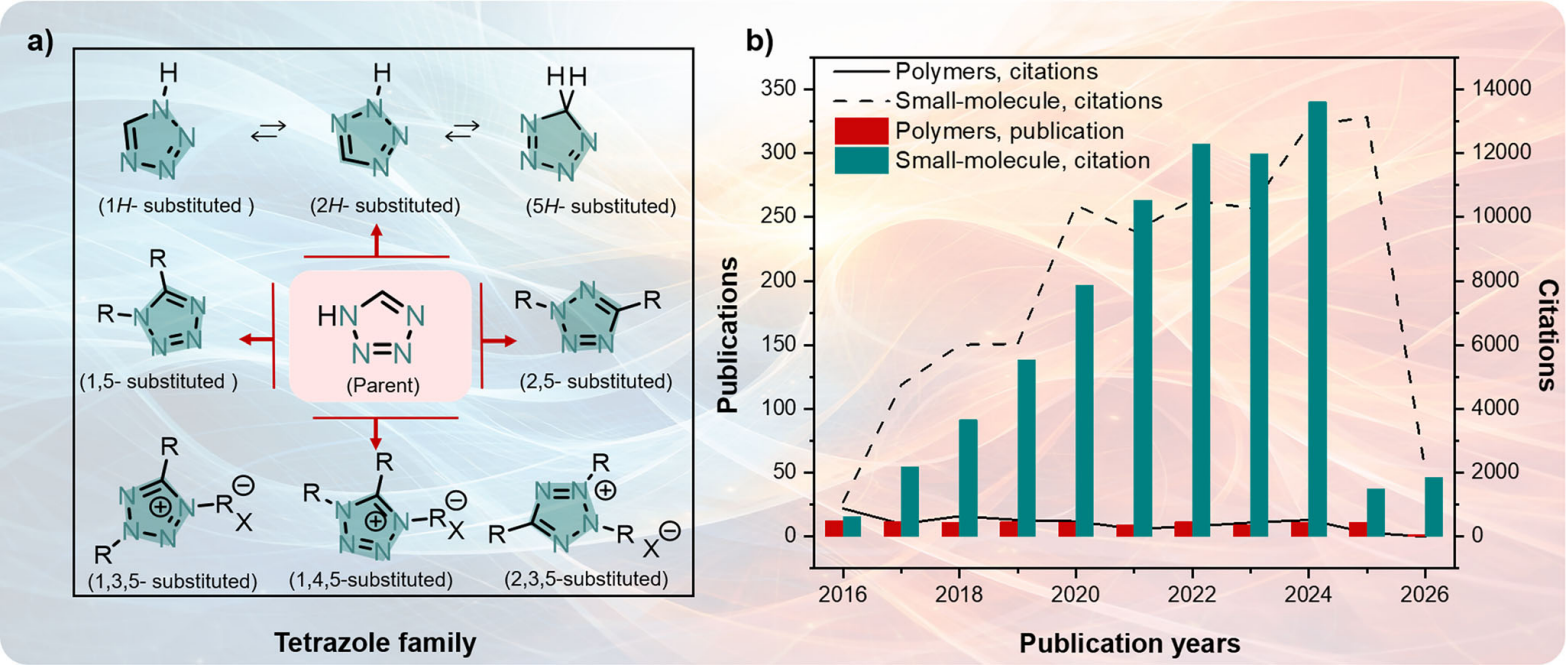
(a) Representative substitution patterns within the tetrazole family, illustrating the structural diversity of the N_4_C heteroaromatic framework (b) Bibliometric analysis of tetrazole‐related research output in polymer chemistry compared with small‐molecule disciplines over the past decade (2016–2026), based on the Scopus database. Annual publication numbers for polymer‐centered studies (red bars) and small‐molecule studies (teal bars) are shown alongside their respective citation trajectories (solid and dashed lines). Tetrazole‐related research in polymer chemistry, identified using the keywords polymer chemistry, coordination polymer, organic polymerization, and aromatic polymer, accounts for 110 publications, 2,166 total citations, and an h‐index of 26. By contrast, tetrazole research in small‐molecule contexts, retrieved using the keywords ligands, energetic materials, metal‐organic frameworks (MOFs), and organometallic coordination, comprises 4,004 publications with 8,442 citations. The data emphasize the substantially greater reasearch activity in small‐molecule tetrazole chemistry compared with polymer‐related tetrazole research. The apparent decline in recent years should be interpreted with caution, as publication and citation data are subject to indexing delays and incomplete coverage in bibliometric databases.

Many functional molecular systems in nature follow a dual design logic: [1, 2, 3] they remain structurally persistent under ordinary conditions while retaining access to alternative functional states that can be activated when change is required. This principle is exemplified by allosteric proteins such as hemoglobin [4, 5, 6, 7], which reversibly switch binding affinity without loss of structural integrity, by gated ion channels that alternate between closed and conductive states in response to defined stimuli [8], and by photoresponsive biomolecular switches such as rhodopsin [9, 10], which remain dormant until optically triggered [11, 12]. In such systems, stability and responsiveness are intrinsically encoded within a single molecular framework through electronic and structural regulation. At a fundamental level, this balance is governed by charge delocalization, ground‐state stabilization, and accessibility of reactive configurations [13, 14, 15, 16, 17, 18, 19, 20]. In this context, tetrazoles provide a particularly instructive synthetic platform. As five‐membered aromatic systems containing four nitrogen atoms (Figure 1a), they combine high heteroatom density with extensive electronic delocalization, giving rise to strongly polarized yet stabilized electronic structures [21, 22]. Despite this well‐defined molecular framework, tetrazole chemistry has developed along markedly different paths across disciplines including medicinal chemistry [23, 24, 25, 26], coordination chemistry [27, 28, 29], energetic materials [30, 31], and photoclick reactions [32, 33, 34]. In these fields, tetrazoles are predominantly treated as discrete functional units optimized for specific binding or reactivity‐ for example bioisosteres [35, 36], coordination ligands [37, 38, 39, 40], or photoreactive precursors [41, 42] in nitrile imine‐mediated tetrazole–ene cycloaddition (NITEC) processes. While this small‐molecule‐centric perspective has enabled broad use, it also limits their consideration of system‐level. For instance, in polymer science, tetrazoles have typically been employed in a utilitarian fashion, valued for their polarity [43, 44, 45] or ion‐binding character [46, 47, 48], yet rarely considered as electronically programmable motifs capable of directing macromolecular behavior. This pragmatic use obscures the connection between the intrinsic electronic structure of tetrazoles and emergent polymer properties, even though polymer architectures inherently amplify molecular‐level features through repetition [49, 50], confinement [51], and cooperative interactions [52, 53]. Despite these advantages, their systematic integration into polymer design remains limited (Figure 1b). A central yet often overlooked factor underlying this disconnect is the substitution pattern, which fundamentally governs the electronic identity and accessible reactivity of the tetrazole ring [22, 54, 55]. Accordingly, tetrazoles can exhibit adaptive [27, 56], triggerable [57], or structurally persistent behavior [58] depending on substitution. While the adaptive and photoresponsive behavior of mono‐ and 2,5‐substituted tetrazoles has been extensively explored in the literature [21, 55, 59, 60, 61], the distinct electronic persistence and structural role of 1,5‐disubstituted tetrazoles remain comparatively underutilized and insufficiently conceptualized, particularly in the context of polymer science. Their integration into macromolecular architectures as electronically defined structural motifs have received only limited attention. Accordingly, this Minireview advances a unifying design framework in which tetrazole substitution pattern dictates electronic identity and, in turn, macromolecular function. Three substitution‐defined regimes, that is, adaptive, triggerable, and structurally persistent, are identified and systematically connected to their manifestation in polymer architectures and their preservation through synthetic strategy. Unlike existing reviews that primarily focus on specific applications or reactivity of tetrazoles, this work explicitly organizes tetrazole chemistry in polymer systems according to substitution‐controlled electronic identity, thereby establishing a unified structure–property framework that links molecular design to macromolecular function. Within this context, particular emphasis is placed on 1,5‐disubstituted tetrazoles as underexplored building blocks for structurally persistent and electronically defined polymer materials.

## 2 The Jekyll–Hyde Paradox: Why Tetrazoles Are Stable Yet Transformable

### 2.1 One Ring, Two Regimes: Aromatic Persistence and Latent Reactivity

**FIGURE 2:**
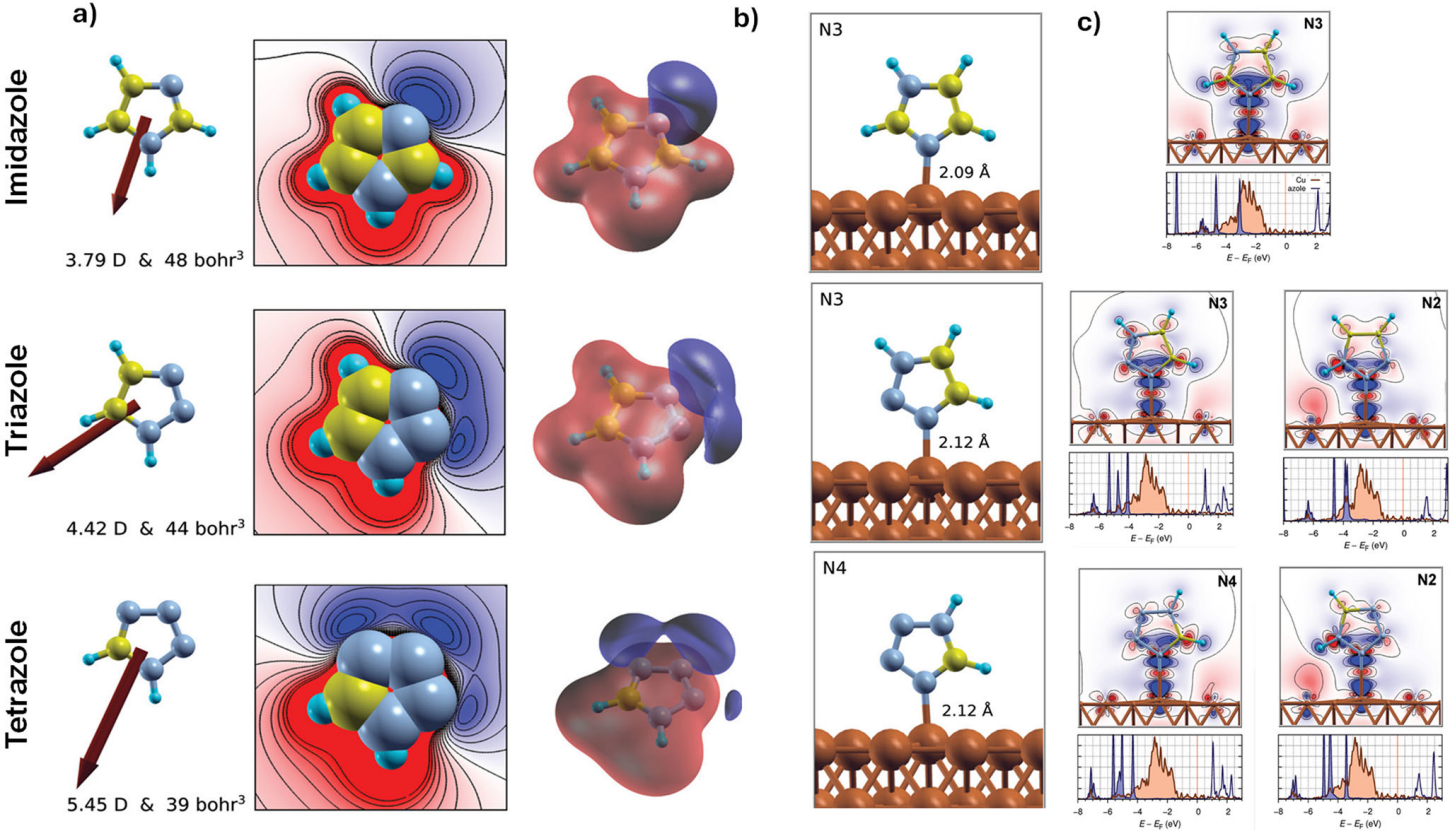
Electronic structure–property relationships across azole systems (imidazole, triazole, and tetrazole). (a) Electrostatic potential maps and dipole moment vectors illustrating the progressive increase in molecular polarization and localization of negative charge at nitrogen sites with increasing heteroatom content. (b) Representative adsorption geometries on Cu(111), showing preferential coordination via nitrogen atoms in an upright configuration, indicating similar adsorption geometries  across the series. (c) Charge density difference (Δ*ρ*) and projected density of states (PDOS) analyses revealing localized charge redistribution upon adsorption and weak σ‐type hybridization with minimal orbital overlap [53]. Reproduced with permission from Ref. [73]. Copyright 2012 American Chemical Society.

Tetrazole systems exhibit dual behavior arising from the distribution of electron density within the N_4_C heteroaromatic ring, which enables both stabilization of the aromatic ground state and access to reactive configurations [62]. This behaviour originates from the high nitrogen content, which modifies both the extent and topology of the π‐electron system [63]. Consequently, the presence of four contiguous nitrogen atoms within a five‐membered aromatic framework leads to a charge distribution that is extensively delocalized yet inherently uneven [64, 65]. The respective electronic structure gives rise to pronounced dipole moments (typically ∼4–6 Debye (D)) [66], placing tetrazoles in a regime where electronic stabilization and responsiveness coexist. As a consequence, the energy gap between the ground state and alternative configurations is reduced, facilitating transitions to reactive states under appropriate conditions [67, 68, 69]. Tetrazole derivatives therefore display pronounced sensitivity to substitution, environment, and external stimuli [70]. The relevant behavior becomes more evident when compared to other five‐membered *N*‐heterocycles. While imidazoles, pyrazoles, and triazoles exhibit aromatic stabilization and substituent‐dependent variation, their electronic responses are more limited due to lower heteroatom density [71, 72]. As illustrated in Figure 2a, increasing nitrogen content from imidazole to tetrazole leads to a systematic rise in dipole moment, accompanied by a progressively stronger localisation of negative electrostatic potential at nitrogen sites, reflecting enhanced molecular polarisation. Despite these pronounced electronic differences, Figure 2b shows that all azoles adopt similar adsorption geometries via *N*–metal coordination, indicating that structural accessibility remains largely unchanged across the series. Consequently, the observed variations in interaction strength originates from intrinsic electronic factors rather than geometric constraints. The latter is further clarified in Figure 2c, where charge density difference and projected density of states analyses reveal that molecule–surface interactions are governed primarily by localised charge redistribution and weak σ‐type hybridisation, with minimal orbital overlap, indicative of limited covalent character [73]. Together, these findings demonstrate that increasing nitrogen content does not enhance covalent interaction but instead shifts the bonding mechanism toward a dipole‐dominated regime. Accordingly, imidazole interacts through localized donor character, triazole represents an intermediate case, whereas tetrazole combines high polarity with increased electronic hardness while maintaining responsiveness primarily through electrostatic interactions [74].

For polymer systems, this implies that increasing nitrogen content primarily enhances dipole‐driven interactions and intermolecular organization rather than covalent bonding strength, an affect that is critical for understanding transport and assembly behavior in tetrazole‐containing materials. For instance, aromatic and heteroaromatic motifs such as phenyl [75] or thiophene [76] typically function as comparatively less reactive structural elements that impart rigidity and stability. Tetrazole‐containing motifs, by contrast, combine both stability and reactivity within the same ring system. The respective duality arises from a unified electronic framework in which persistence and transformability are simultaneously embedded [77]. Quantum chemical studies support this behavior by revealing relatively small energy differences  between the stabilized ground states and activation‐accessible configurations in tetrazole derivatives [78, 79, 80]. Under ambient conditions, delocalization across the nitrogen‐rich ring governs chemical robustness and resistance to hydrolysis, oxidation, and thermal degradation, resulting in a stability comparable to that of stable aromatic systems [62, 81, 82]. Upon activation, the same electronic framework enables access to rearrangements, cycloadditions, and ring‐opening transformations. These processes are frequently driven by the formation of thermodynamically stable small‐molecule products, particularly N_2_, which provides a strong energetic driving force for the transfrormation [83]. All these observations indicate that aromatic stabilization dominates the ground‐state regime, while latent reactivity becomes accessible upon activation through the same electronic framework. Tetrazole derivatives are therefore best understood as, systems poised between persistence and adaptability, providing the molecular basis for their apparent Jekyll–Hyde behavior within a unified energy landscape.

### 2.2 Substitution as the Electronic Switch in Tetrazole Chemistry

While the intrinsic electronic structure of the tetrazole ring encodes both aromatic stabilization and latent reactivity, the balance between these two regimes is not fixed. Instead, it is exquisitely governed by the substitution pattern, which acts as a molecular switch that selectively stabilizes distinct resonance manifolds, tautomeric landscapes, and reaction‐accessible configurations [84, 85]. In this sense, substitution fundamentally defines the electronic identity of tetrazoles [86]. Accordingly, the Jekyll–Hyde analogy reflects the selective expression of either aromatic persistence or adaptive reactivity, depending on substitution and environment. More broadly, this mode of regulation illustrates a general molecular design principles, where small structural changes enable access to different functional states without altering the overall scaffold. In monosubstituted tetrazoles, the presence of a free N‐H proton preserves prototropic tautomerism and enables continuous interconversion between neutral tetrazole and anionic tetrazolate states. This dynamic equilibrium stabilizes charge‐delocalized aromatic forms while simultaneously providing access to acid‐base reactivity and hydrogen‐bond‐mediated interaction networks [87]. These features place monosubstituted tetrazoles near the boundary between stabilized and activated regimes, where environmental factors such as polarity, hydration, and counterion identity can shift the dominant electronic configuration [88, 89]. A distinct behavior emerges in 2,5‐disubstituted tetrazoles, where substitution patterns stabilize excited or activated configurations that facilitate photochemical extrusion of nitrogen and generation of reactive nitrile imine intermediates [90, 91]. Here, aromatic persistence dominates the ground state, but latent reactivity is selectively unlocked under optical activation, enabling rapid cycloaddition reactions widely exploited in tetrazole‐based photoclick chemistry. Experimentally, these transformations are typically initiated under UV irradiation (300–365 nm), with reaction times ranging from seconds to minutes depending on substituent and solvent conditions [92, 93, 94, 95]. This controlled access to high‐energy reaction pathways enables tetrazoles to transition from chemically inert aromatic units to highly reactive species on demand. By contrast, 1,5‐disubstitution progressively suppresses tautomerism and locks the tetrazole ring into electronically fixed aromatic states. In 1,5‐disubstituted tetrazoles, substitution at both the N1 and C5 positions eliminates proton mobility and enforces a neutral heteroaromatic framework with large permanent dipole moments relative to common heteroaromatic units and persistent π‐delocalization [96, 97]. These motifs therefore function as rigid, highly polar structural modules rather than adaptive acid‐base units [98]. Consistent with this behavior, small‐molecule tetrazoles generally undergo thermal decomposition at elevated temperatures, typically initiating above ∼180°C–220°C, and depending strongly on the substitution pattern along the experimental conditions. 1,5‐Disubstituted tetrazoles often exhibit comparatively higher thermal stability due to their persistent aromatic structure, whereas 2,5‐disubstituted derivatives are more prone to N_2_ extrusion upon heating (typically around ∼180°C depending on substituent) [99, 100, 101]. In other words, these substitution‐dependent regimes reveal that the apparent diversity of tetrazole behavior originates from systematic electronic tuning of a single heteroaromatic scaffold. Monosubstituted tetrazoles occupy an adaptive intermediate state, whereas disubstituted derivatives diverge into either electronically locked aromatic modules (1,5‐disubstituted) or optically triggerable reactive precursors (2,5‐disubstituted). Beyond disubstituted frameworks, trisubstituted tetrazoles are accessible through further *N*‐functionalization of 1,5‐disubstituted precursors. However, these species remain electronically locked aromatic heterocycles without prototrophic behavior and thus extend the persistent regime rather than a distinct electronic class [24, 102, 103, 104].

## 3 From Jekyll to Hyde in Macromolecules: Polymer Architecture as Cooperative Amplifier and an Energetic

**FIGURE 3:**
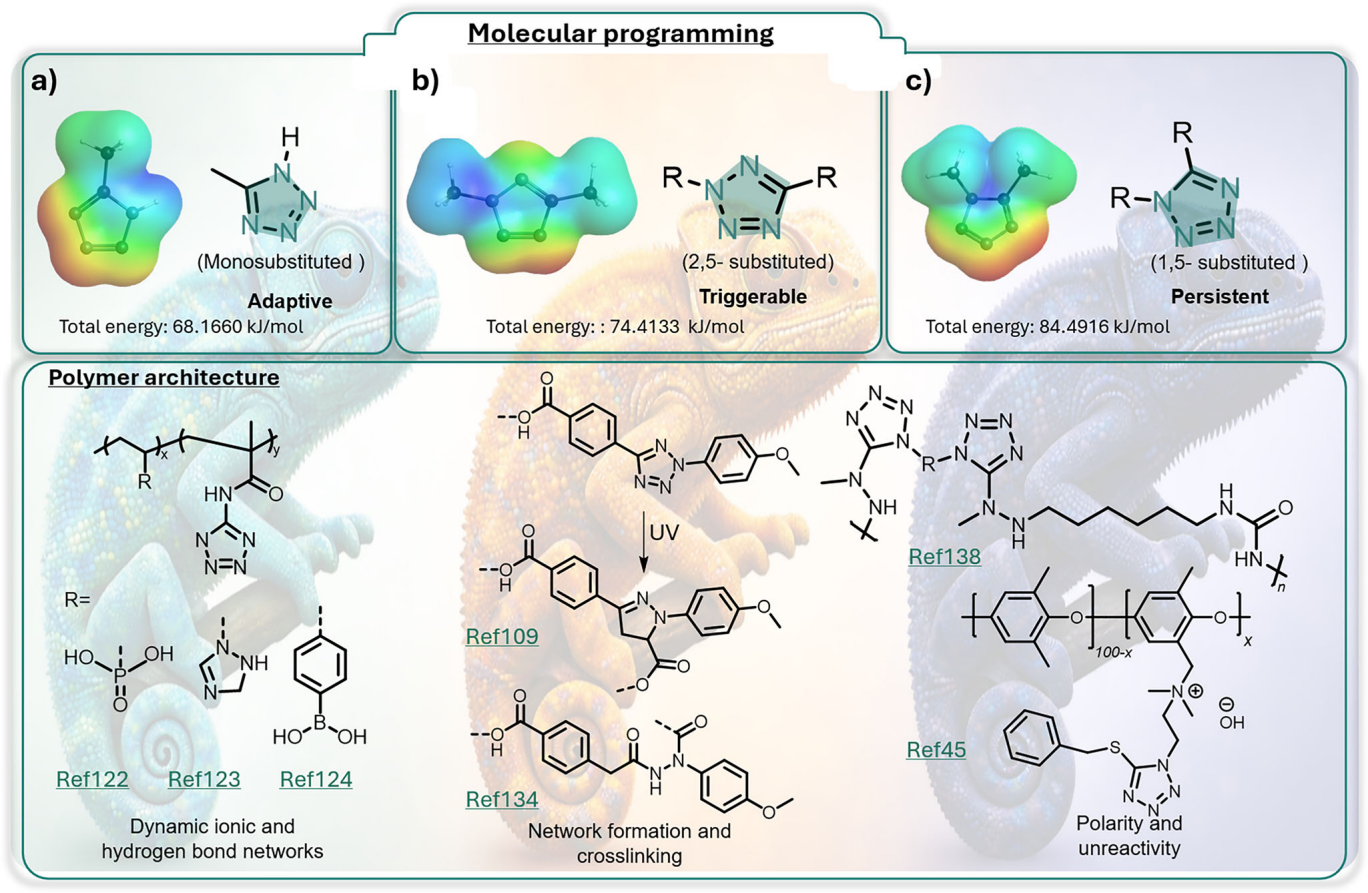
Representative literature examples illustrating substitution‐governed electronic regimes of tetrazoles in polymer systems. (a) Monosubstituted tetrazoles: adaptive tautomeric equilibria enabling dynamic ionic networks. (b) 2,5‐Disubstituted tetrazoles: UV‐triggered nitrile imine formation enabling covalent ligation (i.e., NITEC/NICAL) with fluorescent readout. (c) 1,5‐Disubstituted tetrazoles: electronically locked, persistent heteroaromatic units with enhanced stability. Total energy values estimated using MolView to provide a qualitative comparison of relative energetic stability across the different substitution patterns.

In small molecule chemistry, tetrazoles display an apparent Jekyll–Hyde charactera: on the one hand, they behave as adaptive aromatic units under ambient conditions; on the other hand, they readily undergo activation‐enabled extrusion and rearrangement, frequently driven by N_2_ release [21, 105]. In polymer systems, however, this duality is amplified because chain connectivity, local motif density, and network formation transform intrinsically molecular tetrazole behavior into collective transport [106, 107], ligation [108], and reporting functions. Thus, once tetrazole units are embedded along polymer chains or confined within domains, local dielectric heterogeneity, restricted segmental mobility, and multivalent proximity effects bias the accessible electronic regime [43, 109, 110]. In this sense, polymers do not merely carry tetrazole motifs; they define the microenvironment that selects specific electronic states. This substitution‐governed divergence into adaptive, triggerable, and persistent electronic regimes is schematically summarized in Figure 3 with representative literature examples. As the local concentration of tetrazole units increases, individually weak dipolar, hydrogen‐bonding, and ionic interactions organize into interconnected networks, resulting in cooperative macromolecular behavior [111, 112, 113]. In practice, this amplification is most clearly realized in polymers containing monosubstituted tetrazoles, particularly C5‐substituted derivatives such as 5‐vinyltetrazole (VT) and its polymeric analogue poly(5‐vinyltetrazole) (PVT). In these systems, the presence of a free N‐H functionality enables prototropic tautomerism between neutral tetrazole and anionic tetrazolate forms, establishing a pH‐responsive equilibrium that governs intermolecular interactions [106, 114, 115]. Unlike classical weak polyelectrolytes, where ionization primarily modulates charge density, tetrazole units introduce a heteroaromatic, delocalized charge state that stabilizes the tetrazolate form and enables more directional and cooperative ionic interactions [116]. Experimental studies on PVT show that partial deprotonation leads to tetrazolate formation and promotes interchain ionic association, resulting in polyelectrolyte‐like behavior [106]. Quantitatively, this behavior is reflected in proton conductivities of up to ∼10^−^^3^ S·cm^−^^1^ under optimized humidity and temperature conditions, accompanied by controlled swelling (∼50%–200%), consistent with transient ionic junction formation [115]. Importantly, the properties of tetrazole‐containing polymers are not only intrinsic but also tunable through composition, as demonstrated by Pu et al., where increasing tetrazole content systematically enhances proton conductivity (from ∼10^−6^ to 10^−3^ S·cm^−^^1^) and reinforces intermolecular interactions. This establishes a direct structure‐property relationship, in which tetrazole density governs ionic network formation and, consequently, macroscopic transport [107]. When benchmarked against conventional solid polymer electrolytes, tetrazole‐containing polymers exhibit competitive or superior ionic transport. Typical systems such as poly(ethylene oxide)‐lithium salt complexes display conductivities of ∼10^−^^7^ S·cm^−^^1^ [117], while blended systems such as poly(ethylene oxide)/poly(vinylpyrrolidone) reach ∼1.14 × 10^−5^ S·cm^−^^1^ [118]. More advanced polymer electrolytes, including polycarbonates (∼4 × 10^−5^ S·cm^−^^1^) and polysiloxanes (∼3 × 10^−4^ S·cm^−^^1^), remain within the same order‐of‐magnitude regime [119]. In comparison, PVT systems achieve conductivities up to ∼10^−^^3^ S·cm^−^^1^, highlighting the intrinsic contribution of tetrazolate formation to ionic transport. Moreover, compositional tuning provides an additional level of control: increasing tetrazole content in copolymer systems systematically enhances proton conductivity (from ∼10^−^^6^ to 10^−^^3^ S·cm^−^^1^), establishing a direct structure‐property relationship between tetrazole density and ionic network formation [107]. For instance, in tetrazole‐functionalized chitosan systems reported by Egorov et al., increasing the degree of substitution (0.10, 0.30 and 0.65) promotes a transition from interfacial activity to catalytic dominance, with nanoparticle formation at low substitution (∼30 nm, ζ ≈ +48 mV) and catalytic performance increasing from 0% conversion in native chitosan to ∼100% within 60 min, reaching quantitative conversion in 15 min at high substitution levels [120]. Studies from Sinirlioğlu et al. demonstrate that tetrazole‐based polymers significantly exceed these limitations when appropriately integrated into copolymers or doped systems. For example, incorporation of acidic vinyl phosphonic acid units into poly(5‐methacryltetrazole) increases proton conductivity from ∼2 × 10^−^^6^ S·cm^−^^1^ in the homopolymer to ∼10^−^^2^ S·cm^−^^1^ at 150°C, accompanied by reduced glass transition temperatures and suppression of crystallinity, indicating enhanced chain mobility and hydrogen‐bond network formation [121]. Similarly, tetrazole‐triazole copolymers doped with phosphoric acid exhibit conductivities of ∼0.012–0.016 S·cm^−^^1^ under anhydrous conditions, where proton transport is facilitated by combined heterocyclic proton hopping and phosphate‐mediated pathways [122]. Further enhancement is observed in boronic acid–tetrazole systems, where synergistic acid‐base interactions enable conductivities up to ∼0.021 S·cm^−^^1^ at elevated temperatures, although at the expense of reduced thermal stability at high doping levels [123]. Complementary trends are also evident in azole‐functionalized methacrylate systems, where tetrazole‐containing polymers reach ∼0.01 S·cm^−^^1^ upon acid doping, confirming that proton transport efficiency is strongly dependent on both functional group identity and acid‐base interaction strength [124]. Taken together, these results demonstrate that tetrazole polymers exhibit intrinsic proton conductivity that is significantly enhanced in multicomponent or doped systems through the formation of extended, cooperative hydrogen‐bonding networks. In monosubstituted tetrazole systems, transport is dictated by protonation‐deprotonation equilibria, where the tetrazole/tetrazolate pair enables hydrogen‐bond‐assisted ion hopping. Tetrazole‐functionalized membranes reported by Henkensmeier and coworkers exhibit hydroxide conductivities of ∼10‐20 mS cm^−^^1^ at 60°C–80°C, outperforming imidazole‐based systems (∼1‐5 mS cm^−^^1^). However, the reliance on dynamic equilibria introduces an inherent limitation, as random distribution and environmental sensitivity lead to electronic heterogeneity and reduced predictability [125, 126]. A shift from adaptive to fixed ionic behavior is achieved through N‐alkylation, forming tetrazolium derivatives. In a representative system, Kulkarni et al. developed a polyacrylonitrile (PAN)‐based gel electrolyte exhibiting a conductivity of 1.67 mS cm^−^^1^ and a lithium‐ion transference number of t_Li_^+^ ≈ 0.90, approaching single‐ion conduction due to strong anion immobilization [127]. This behavior is supported by spectroscopic evidence showing reduced anion mobility. Compared with conventional polyethylene oxide (PEO)‐based electrolytes (t_Li_^+^ ≈ 0.2–0.4), these systems demonstrate substantially more decoupled ion transport [128]. Beyond ionic and adaptive behavior, the same nitrogen‐rich tetrazole framework enables energetic functionality. In PVT polymers, thermal decomposition occurs at ∼220°C–300°C with exothermic enthalpies of ∼1.5–3.5 kJ g^−^^1^, driven by nitrogen evolution [129]. Compared with glycidyl azide polymer (GAP), which decomposes at ∼180°C–220°C, tetrazole‐based systems offer improved thermal stability and reduced sensitivity. Polymer chain connectivity further distributes energy release spatially, enabling controlled energetic performance in propellants and gas‐generating systems [130].

A fundamentally different behavior is observed for 2,5‐disubstituted tetrazoles, particularly aryl‐substituted diphenyltetrazole derivatives. In contrast to monosubstituted systems, these compounds do not act as adaptive ionic motifs but instead serve as photoactive units that convert light directly into covalent bond formation. This reactivity is typically based on nitrile imine‐mediated tetrazole–ene cycloaddition (NITEC) or nitrile imine carboxylic acid ligation (NICAL) reactions. Upon UV irradiation (300–365 nm), the tetrazole releases N_2_ to generate a nitrile imine intermediate, which reacts rapidly with enes, maleimides, or carboxylic acids, while forming a fluorescent pyrazoline product. The direct link between bond formation and fluorescence makes these systems especially useful as self‐reporting alternatives to conventional photo‐crosslinkers [131]. Importantly, tetrazole photolysis is not strictly limited to the absorption maximum of the chromophore. Photochemical action‐plot studies have demonstrated that UV/vis absorption spectra alone do not necessarily predict wavelength‐dependent photochemical reactivity, with productive reactivity often extending to wavelengths red‐shifted from the absorption maximum [132]. This behavior is particularly relevant for tetrazole‐based photochemical–thermal cascades, where photoexcitation induces N_2_ extrusion to generate nitrile imines that subsequently undergo 1,3‐dipolar cycloaddition with suitable dipolarophiles. In wavelength‐dependent NITEC studies, 2,5‐diphenyltetrazoles were found to react most efficiently below 295 nm but could still be employed for photoligation up to 322 nm [133]. More strongly red‐shifted systems have been achieved through chromophore engineering: extended π‐conjugation enabled tetrazole activation at 405 nm, pyrene–tetrazole systems at 420 nm, and dimethylamino‐pyrenyl aryl tetrazoles up to 515 nm [134]. In polymer materials, *N*,*N*‐dimethylamino‐pyrenyl aryl tetrazole (APAT)‐based tetrazole chromophores have further enabled λ‐orthogonal NITEC reactions at 450 nm for cooperative network formation [135]. Beyond direct one‐photon excitation, two‐photon excitation at 700 nm and upconversion‐assisted strategies using 974 nm near‐infrared irradiation have also been used to trigger NITEC chemistry [42]. This wavelength‐dependent behavior is particularly valuable for spatially controlled synthesis, polymer modification, materials patterning, and multi‐wavelength materials design. One of the earliest clear examples was reported by Dietrich et al. [136], who used 2,5‐disubstituted tetrazoles as photoclick handles for polymer conjugation and surface grafting. Tetrazole–polyethylene glycol (PEG) and maleimide–PEG formed diblock structures within 10–15 min under 254 nm irradiation. The same approach enabled poly(methyl methacrylate) (PMMA) to be grafted onto silicon and cellulose surfaces. Notably, the modified cellulose became strongly hydrophobic (water contact angle 126°), whereas the starting material showed no such behavior. Thus, the tetrazole acts as a photochemical trigger for the controlled installation of functional groups rather than as the final functional element. Similar behavior is observed in particulate systems. Wang et al. incorporated 2,5‐disubstituted tetrazoles into step‐growth microparticles (average diameter 4.1 µm, CV = 12%), where the NITEC reaction reached full conversion within 10 min under 320 nm irradiation. The fluorescence intensity increased with tetrazole content (129–354 a.u. from 2.5–7.5 mol%), while the glass transition temperature changed only slightly (by only 1°C–4°C), indicating that these units mainly contribute to ligation and optical response rather than mechanical properties [137]. Hooker et al. used the same chemistry directly as a crosslinking step to form fluorescent microspheres within 15 min under mild UV conditions. Compared with conventional polymerization methods, which often require more than 24 h, this chemistry provides a fast and efficient route to structured materials with built‐in optical readout [138]. At smaller length scales, Molle et al. showed that tetrazole content also affects self‐assembly. Increasing the amount of tetrazole shifts the morphology from spherical particles to fibers and vesicle‐like structures. At the same time, the tetrazole units remain reactive, allowing further modification through NITEC reactions at the particle surface [139]. In other words, these studies demonstrate that 2,5‐disubstituted tetrazoles act as light‐controlled functional units that enable fast covalent bond formation, structural control, and simultaneous fluorescence readout. They are therefore particularly attractive for designing polymer systems requiring spatial and temporal control over structure and function.

### 3.1 Precision Motifs Emerge: The Ascendancy of 1,5‐Disubstituted Tetrazoles

While monosubstituted tetrazoles show adaptive supramolecular behavior and 2,5‐disubstituted derivatives enable phototriggered reactions, both are limited in different ways, either by their strong environmental dependence or by irreversible conversion upon activation. In contrast, 1,5‐disubstituted tetrazoles offer an electronically persistent alternative. Substitution at the N1 and C5 positions suppresses prototropic tautomerism and removes photochemical reactivity, leaving a neutral and electronically well‐defined heteroaromatic unit that can be used as a stable structural element rather than a reactive group [98]. Early evidence for this structural persistence comes from energetic polymer research. Sproll and Klapötke showed that incorporating tetrazole units into polymer backbones leads to materials with decomposition temperatures of ∼174°C–276°C, exceeding those of nitrate ester‐based systems (∼170°C–175°C) and with improved handling relative to azide‐containing polymers such as glycidyl azide polymer (GAP) [140, 141]. Similar trends were reported by Hartdegen, who found decomposition temperatures in the range of ∼200°C–270°C, along with reduced impact and friction sensitivity compared with nitrate ester and azide‐based systems [142]. More recent work has expanded the use of 1,5‐disubstituted tetrazoles beyond energetic materials. In alkaline anion‐exchange membranes, Bakangura et al. incorporated these units into poly(phenylene oxide) (PPO), achieving hydroxide conductivities of 14.7–23.2 mS cm^−^^1^ at relatively low ion‐exchange capacities (IEC ≈ 1.11–1.32 mmol g^−1^ ol g^−^^1^). At the same time, the activation energy for ion transport decreased from 16.99 to 8.3 kJ mol^−^^1^ compared with conventional quaternized PPO systems, indicating more efficient transport pathways. These results suggest that 1,5‐disubstituted tetrazoles can stabilize ion‐conducting networks while limiting excessive swelling, a common limitation of polyelectrolytes [45]. This behavior is further supported by our recent work, which demonstrated the tetrazole unit remains intact under thiol–ene polymerization conditions in contrast to 2,5‐disubstituted analogues, which undergo photochemical transformation under similar conditions. Thermal analysis confirmed stability up to ∼300°C, with tunable glass transition temperatures (*T*_g_ = −43°C to −13°C), highlighting the robustness of these structures [98]. The respective stability is also retained in main‐chain architectures, for which we reported 1,5‐disubstituted tetrazole‐containing polymers with tunable glass transition temperatures (*T*_g_ = −33°C to 21°C) and decomposition temperatures of ∼300°C–311°C. The corresponding materials exhibit thermal stability comparable to that of conventional polyamides, including PA6 and PA66, while offering greater flexibility in structural design [143]. Taken together, the behavior of tetrazoles in polymer systems is governed by the interplay between substitution pattern and macromolecular environment. The same heterocycle can support adaptive interactions, undergo photochemical transformation, or remain structurally persistent, depending on how it is incorporated. These regimes can therefore be selectively accessed through molecular design and polymer architecture. In this context, the often‐described “Jekyll–Hyde” character of tetrazoles becomes a controllable feature that enables targeted tuning of polymer properties.

## 4 Synthesis as a Design Gatekeeper: Preserving Tetrazole Electronic Identity

### 4.1 Direct Monomer Polymerization: Transferring Adaptive Tetrazoles into Polymer Side Chains

**FIGURE 4:**
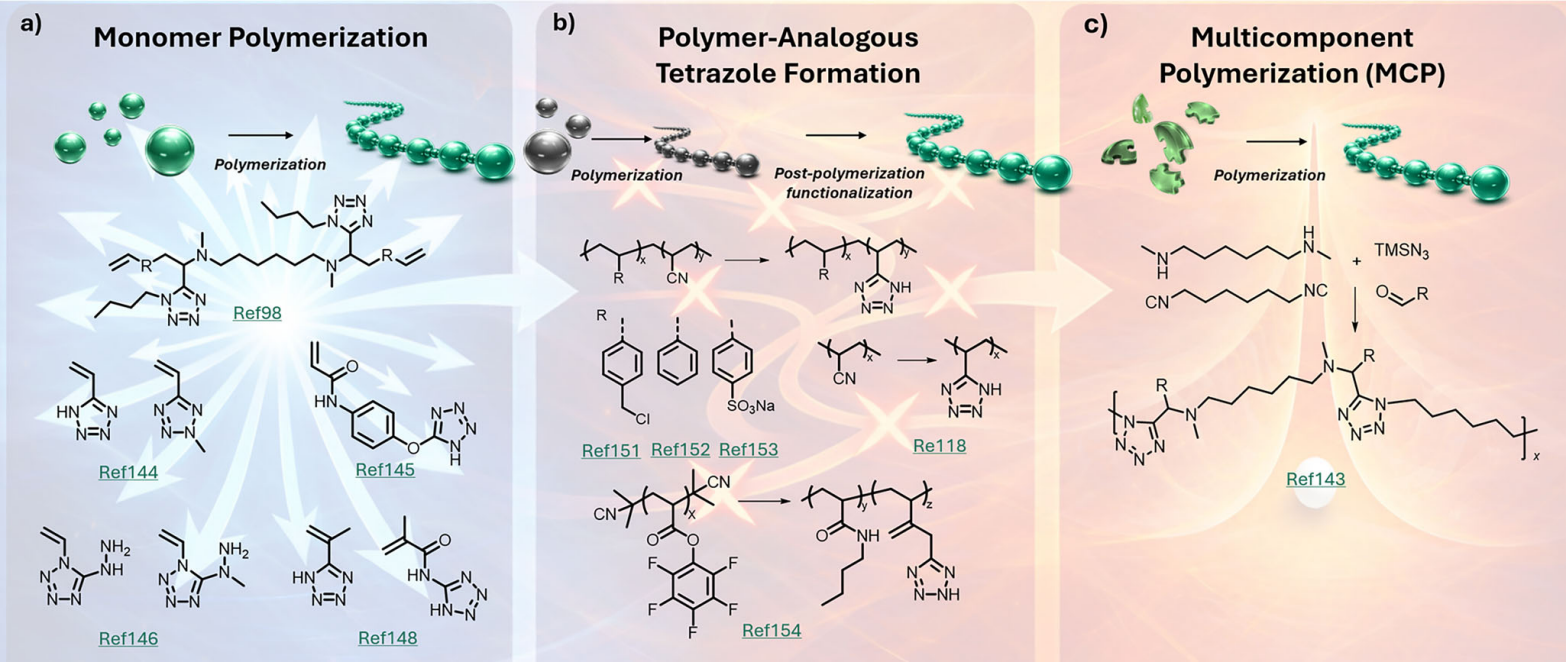
Synthetic routes to tetrazole‐containing polymers and representative literature implementations. (a) Representative tetrazole‐containing monomers reported in the literature for direct polymerization. (b) Polymer‐analogous post‐polymerization modification strategies leading to tetrazole‐containing polymers, with representative literature examples. (c) Multicomponent polymerization (MCP) enabling the formation of main‐chain tetrazole units, illustrated by the only reported example to date.

Direct polymerization of tetrazole‐bearing monomers represents the earliest and most straightforward strategy for constructing tetrazole‐containing macromolecules. Vinyl tetrazoles and related unsaturated derivatives have been synthesized and polymerized via conventional free‐radical routes, as well as copolymerized with styrenic and acrylate comonomers to tune tetrazole content and material properties [141, 144, 145]. In these systems, the tetrazole motif is introduced in its monosubstituted form, preserving prototropic tautomerism and moderate acidity [146, 147, 148] Representative tetrazole‐bearing monomers reported in the literature are illustrated in Figure 4a.

### 4.2 Polymer‐Analogous Tetrazole Formation: Converting Static Backbones into Dynamic Platforms

Post‐polymerization transformations of nitrile functionalities into tetrazoles offer a conceptually distinct yet functionally analogous strategy. Through azide‐nitrile cycloaddition, commodity polymers such as polyacrylonitrile can be efficiently transformed into poly(5‐vinyltetrazole) and related tetrazole‐rich materials [149, 150, 151, 152]. This approach decouples backbone synthesis from tetrazole installation, enabling access to well‐defined polymer architectures prior to functional transformation. High degrees of conversion and scalability have made this route particularly attractive for membrane materials and *N*‐rich polymer systems. Nevertheless, the resulting behavior mirrors that of vinyl tetrazole polymerization: monosubstituted tetrazoles dominate, retaining tautomeric flexibility and acid‐base responsiveness. As a result, these polymers function as dynamic ionic platforms whose macroscopic properties remain strongly coupled to processing conditions and their chemical environment. Figure 4b summarizes representative nitrile‐to‐tetrazole conversion strategies alongside the corresponding polymer examples, highlighting how static precursor backbones can be transformed into tetrazole‐rich materials. Beyond nitrile‐based transformations, alternative post‐polymerization strategies employing activated ester platforms further expand the synthetic toolbox while, largely retaining the adaptive behavior of monosubstituted tetrazoles. In particular, pentafluorophenyl ester‐containing polymers can be efficiently transformed via nucleophilic substitution with tetrazole derivatives, enabling modular installation of tetrazole units under mild conditions with high functional group tolerance. The additional examples shown in Figure 4b, further highlight that polymer‐analogous approaches broaden structural accessibility while preserving the characteristic adaptive behavior of monosubstituted tetrazoles [153, 154, 155].

### 4.3 Multicomponent Polymerization (MCP): From Functional Group Incorporation to Electronic Programming

Multicomponent polymerization (MCP) has emerged as a powerful synthetic strategy in polymer chemistry, enabling the simultaneous formation of multiple covalent bonds in a single step while offering high modularity and structural diversity [156]. By integrating three or more building blocks in one reaction event, MCP allows the direct encoding of functional motifs during macromolecular assembly, rather than through post‐synthetic modification [157, 158]. In the context of tetrazole‐containing polymers, this strategy has only recently been translated to macromolecular systems. The Ugi–azide multicomponent reaction enables the formation of 1,5‐disubstituted tetrazole units within polymer structures, thereby defining the substitution pattern at the moment of bond formation. Studies demonstrate that this approach can be applied both to monomer synthesis [98] and main‐chain polymer synthesis [140], affording structurally defined tetrazole‐containing polymers in a single step while preserving the integrity of the heteroaromatic unit throughout the polymerization process. Despite its proven efficiency in small‐molecule chemistry, the application of MCP to tetrazole‐based polymer systems remains comparatively limited, with only a few representative studies reported to date. Nevertheless, these studies highlight the considerable potential of MCP for accessing electronically defined tetrazole architectures that are difficult to obtain by conventional methods.

## 5 Conclusion

Tetrazoles are often treated as a single functional group in polymer chemistry; however, they are more appropriately considered a family of functionally distinct heterocycles whose behavior is governed by substitution pattern and influenced by synthetic strategy. The apparent Jekyll–Hyde character of tetrazoles can thus be understood not as a contradiction, but as a substitution‐dependent differentiation of a single aromatic framework into three regimes: adaptive, triggerable, and persistent. Within this framework, monosubstituted tetrazoles function as adaptive motifs that enable environmentally responsive hydrogen bonding and ionic reorganization, whereas 2,5‐disubstituted derivatives act as photoaddressable units capable of controlled activation within polymer systems. In contrast, regio‐defined 1,5‐disubstituted tetrazoles represent structurally persistent motifs that promote persistent polarity, structural cohesion, and predictable material behavior. These regimes are not interchangeable, and distinguishing between them is essential for establishing meaningful structure–property relationships in tetrazole‐based materials. Synthetic strategy plays a central role in determining which regime is realized. Approaches that enable regioselective incorporation, such as multicomponent reactions, facilitate the preservation of well‐defined substitution patterns during polymer formation. In contrast, non‐selective or mixed substitution patterns may lead to averaged or ill‐defined materials properties, thereby limiting predictability. In combination, substitution pattern, synthetic control, and polymer architecture act as interdependent design parameters that determine material performance. More broadly, tetrazoles illustrate how molecular design can be translated into predictable macroscopic properties. Accordingly, substitution‐guided molecular design may enable a more systematic and predictive use of tetrazoles‐and related heterocycles in polymer materials. In other words, from this perspective, three practical design guidelines could be emerged: (i) monosubstituted tetrazoles should be selected when adaptive ionic or hydrogen‐bonding networks are desired, (ii) 2,5‐disubstituted tetrazoles are suitable for photo‐triggered ligation and spatially controlled functionalization, and (iii) 1,5‐disubstituted tetrazoles provide structurally persistent, defined motifs for stable polymer architectures.

## 6 Outlook: Toward a Substitution Defined Design Standard for Tetrazoles in Polymer Science

The perspective developed in this mini‐review shows that tetrazole chemistry in polymer science is still not used in a fully consistent way. While the electronic duality of tetrazoles is well understood in small‐molecule chemistry, its translation into polymer design remains incomplete. A main challenge is the lack of precise control over substitution patterns in polymer systems. Many current synthetic approaches lead to mixtures or ill‐defined structures, making it difficult to establish clear structure ‐property relationships. Developing more selective and scalable synthetic methods, especially for main‐chain and sequence‐defined polymers, will therefore be important.

At the same time, the role of the polymer environment is poorly understood. Factors such as polarity, confinement, and chain mobility clearly influence tetrazole behavior, but they are rarely studied in a systematic way, making reliable prediction of material performance difficult. Another open point is the combination of different tetrazole functions within one material. Most systems so far rely on only one type of behavior, while combining adaptive, photoresponsive, and persistent units in a controlled way is still largely unexplored. Such approaches could be useful for materials that need both stability and responsiveness, for example in membranes or coatings.

From a synthetic side, multicomponent polymerization and post‐polymerization modification offer useful routes, as they allow tetrazole units to be introduced at defined stages, and in some cases with better control over substitution. These methods still require further development, especially for more complex and scalable polymer architectures. In addition, new tools such as data‐driven methods, machine learning, and high‐throughput screening offer opportunities to accelerate understanding of structure–property relationships and support material design, especially in systems where multiple variables operate simultaneously.

Finally, tetrazole‐containing polymers are still not widely used in areas where their properties could be useful, such as ion‐conducting materials, membranes, or systems that require controlled activation. Progress in these areas will depend on linking molecular design more directly to application performance. As Stevenson wrote, “*man is not truly one, but truly two*,” [159] and in a similar way, tetrazoles do not represent conflicting behaviors but a controlled duality that can be selected and directed through molecular design. What is needed now is the deliberate integration of  synthetic control, mechanistic understanding, and targeted application design to turn these systems into reliable material platforms.

## Author Contributions

Meryem S. Akdemir: conceptualization, writing of the original draft, review and editing of the manuscript. Hatice Mutlu: conceptualization, writing of the original draft, review and editing of the manuscript, supervision, funding acquisition.

## Conflicts of Interest

The authors declare no conflicts of interest.

## Data Availability

Data sharing is not applicable to this article, as no datasets were generated or analyzed during the current study.
